# Resveratrol Improves Boar Sperm Quality via 5′AMP-Activated Protein Kinase Activation during Cryopreservation

**DOI:** 10.1155/2019/5921503

**Published:** 2019-09-04

**Authors:** Zhendong Zhu, Rongnan Li, Xiaoteng Fan, Yinghua Lv, Yi Zheng, S. A. Masudul Hoque, De Wu, Wenxian Zeng

**Affiliations:** ^1^Key Laboratory of Animal Genetics, Breeding and Reproduction of Shaanxi Province, College of Animal Science and Technology, Northwest A&F University, Shaanxi 712100, China; ^2^Department of Animal Breeding and Genetics, Bangabandhu Sheikh Mujibur Rahman Agricultural University, Gazipur 1706, Bangladesh; ^3^Key Laboratory for Animal Disease Resistance Nutrition of the Ministry of Education of China, Institute of Animal Nutrition, Sichuan Agricultural University, 211 Hui-min Road, Wenjiang, Chengdu, Sichuan 611100, China

## Abstract

Mammalian sperm is highly susceptible to the reactive oxygen species (ROS) stress caused by biochemical and physical modifications during the cryopreservation process. 5′AMP-activated protein kinase (AMPK) is involved in regulating both cell metabolism and cellular redox status. The aim of the present study was to investigate whether the resveratrol protects boar sperm against ROS stress via activation of AMPK during cryopreservation. Boar sperm was diluted with the freezing medium supplemented with resveratrol at different concentrations (0, 25, 50, 75, 100, and 125 *μ*M). It was observed that the addition of 50 *μ*M resveratrol significantly improved the postthaw sperm progressive motility, membrane integrity, acrosome integrity, mitochondrial activity, glutathione (GSH) level, activities of enzymatic antioxidants (glutathione peroxidase (GPx), superoxide dismutase (SOD), and catalase), and the phosphorylation of AMPK. Meanwhile, the lipid peroxidation, ROS levels, and apoptosis of postthaw sperm were reduced in the presence of 50 *μ*M resveratrol. Furthermore, when fresh boar sperm was incubated with the medium in the presence of 50 *μ*M resveratrol and 30 *μ*M Compound C (an AMPK inhibitor), the effects of the resveratrol were partly counteracted by the Compound C. These observations suggest that the resveratrol protects boar sperm via promoting AMPK phosphorylation. In conclusion, the addition of resveratrol to the freezing extenders protects boar sperm against ROS damage via promoting AMPK phosphorylation for decreasing the ROS production and improving the antioxidative defense system of postthaw sperm. These findings provide novel insights into understanding the mechanisms of resveratrol on how to protect boar sperm quality contrary to the ROS production during cryopreservation.

## 1. Introduction

The cryopreservation of sperm is one of the most essential assisted reproductive techniques in the livestock industry. This technique involves some physiochemical procedures including cooling, freezing, and thawing, which are known to produce excess ROS through an alteration in the sperm physical or chemical conditions that impair the homeostasis of sperm metabolism [1]. The removal of seminal plasma during the cryopreservation process is reported to reduce the antioxidant defenses in sperm [2–4], and thus, sperm becomes vulnerable to the oxidative stress [5]. Boar sperm plasma membrane is rich in polyunsaturated fatty acids with low cholesterol or phospholipid that are susceptible to lipid peroxidation by ROS attack *in vitro* [6]. The excessive ROS generated during the cryopreservation process is detrimental to sperm motility and fertilizing ability [7]. Numerous studies reported that the treatment with exogenous antioxidants is an effective strategy to resist oxidative stress and to improve sperm quality during cryopreservation [8–10].

Resveratrol (3,5,4′-trihydroxystilbene), a stilbenoid, is a natural polyphenol structurally similar to diethylstilbestrol and estradiol [11]. Resveratrol was first identified as the principal active ingredient from the dried roots of *Polygonum cuspidatum*, a plant used in traditional Chinese medicine for responding to injury, stress, bacterial or fungal infection, UV irradiation and exposure to ozone [12, 13]. Previous studies showed that resveratrol is widely consumed in the Mediterranean diet in the form of grapes, peanuts, and red wine [14, 15]. Additionally, the resveratrol is the main reason for the “French paradox” that French people who had a low incidence of coronary heart diseases despite the consumption of a diet with high saturated fat [16]. In somatic cells, resveratrol has been regarded as an antioxidant due to its ability to reduce mitochondrial ROS production, scavenge superoxide radicals, and inhibit lipid peroxidation as well as regulate the expression of antioxidant cofactors and enzymes [17]. Furthermore, *in vivo* studies revealed that resveratrol plays a beneficial role against the diseases of aged people having impaired energy metabolism [15]. Adenosine triphosphate (ATP) production through the mitochondrial oxidative phosphorylation (OXPHOS) is essential to maintain motility tracks in boar sperm [18]. However, ROS generation is a natural by-product of mitochondrial OXPHOS [19, 20]. The ROS level in sperm is controlled by the balance between ROS generation and ROS scavenging by antioxidants [21]. Notably, AMPK is an energy sensor of cellular metabolism [22] that was observed to be activated by resveratrol in somatic cells *in vitro* [23, 24]. Additionally, our previous study has been identified that the AMPK regulates energy metabolism in goat sperm [25]. Therefore, it is hypothesized that the addition of resveratrol to the freezing extender may activate the AMPK in sperm. If the AMPK is activated by resveratrol in sperm, it will not only scavenge the ROS but also enhance the sperm antioxidative defense system.

Numerous studies have been reported that the addition of resveratrol to the freezing extenders improves sperm motility and mitochondrial activity in bulls [26] and reduces the DNA damage of postthaw human sperm [27]. Additionally, Gadani et al. reported that the addition of resveratrol to the thawing solution efficiently improved the penetration rate of boar sperm *in vitro* [28]. However, there is no report on the addition of resveratrol to the freezing extender of boar sperm. Moreover, the mechanism of how the resveratrol protects sperm against oxidative damages is still unclear. Therefore, this study was aimed at investigating the effect of resveratrol treatment on boar sperm during cryopreservation and at understanding the mechanism of how resveratrol protects boar sperm against ROS attack. In this study, we hypothesized that the resveratrol might protect the sperm against ROS-associated damages by reducing the ROS generation through the activation of AMPK and consequently enhance the sperm antioxidant systems during cryopreservation.

## 2. Experiment Design

Experiment I was designed to detect whether the addition of the resveratrol to the freezing extender could improve the quality of frozen-thawed boar sperm via examination of sperm progressive motility, membrane integrity, intact acrosome, mitochondrial activity, oxidative DNA damage, and lipid peroxidation. The ROS level, GSH content, activities of GPx, SOD, and catalase, and apoptosis as well as AMPK phosphorylation were also analyzed to reveal the mechanism in which resveratrol protects sperm via activating AMPK and how it enhances sperm's antioxidative defense system.

Experiment II was set up to study whether resveratrol could protect sperm during the process of cryopreservation from cooling, equilibration, freezing, and thawing and incubation of frozen-thawed sperm *in vitro*.

Experiment III was carried out to verify that the role of resveratrol in protecting boar sperm via activating AMPK against ROS stress *in vitro*. H_2_O_2_ was used to induce ROS damage; AMPK activator (5-aminoimidazole-4-carboxamide-1-beta-4-ribofuranoside (AICAR)) and inhibitor (Compound C) were used to regulate AMPK. Specifically, as shown in Supplementary [Supplementary-material supplementary-material-1], there were five treatment groups in Experiment III, where the Modena extender was supplemented with 200 *μ*M H_2_O_2_ (1), with 200 *μ*M H_2_O_2_ and 50 *μ*M resveratrol (2), with 200 *μ*M H_2_O_2_ and 2 mM AICAR (3), with 200 *μ*M H_2_O_2_, 50 *μ*M resveratrol, and 30 *μ*M Compound C (4), and Modena extender without resveratrol, AICAR, Compound C, or H_2_O_2_ (5). Sperm progressive motility, acrosome integrity, membrane integrity, mitochondrial activity, lipid peroxidation, GSH level, ROS, and activities of GPx, SOD, and catalase, along with AMPK phosphorylation, were analyzed in those treatments.

## 3. Materials and Methods

### 3.1. Chemicals and Extenders

All chemicals and reagents were purchased from Sigma-Aldrich, China, unless specified otherwise.

The Modena solution was prepared in the laboratory which composed of 153 mM D-glucose, 26.7 mM trisodium citrate, 11.9 mM sodium hydrogen carbonate, 15.1 mM citric acid, 6.3 mM EDTA-2Na, 46.6 mM Tris, 1000 IU/mL penicillin G sodium salt (Solarbio, Beijing, China), 100 *μ*g/mL polymyxin B, and 1 mg/mL streptomycin sesquisulfate (Solarbio, Beijing, China). Modena solution was used as the extender for liquid semen or thawing solution. The freezing extender (NSF) as described by Okazaki et al. [29] was used in this study with some modifications (mNSF). The first modification was done by adjusting the osmolarity to 400 mOsm/kg at a final concentration (mNSF1). The mNSF1 was further modified as mNSF2 by adding 1.5% (*v*/*v*, final concentrations: 0.75%) Orvus Es Paste (Miyazaki Chemical Sales Ltd., Tokyo, Japan) and 4% (*v*/*v*, final concentrations: 2%) glycerol.

In Experiment I, resveratrol was added to the mNSF1 at concentrations of 0, 25, 50, 75, 100, or 125 *μ*M to determine the optimum concentration, which was used to the mNSF1, mNSF2, or Modena solution in the subsequent Experiments II and III.

### 3.2. Collection of Semen

Seven mature and fertile Duroc boars (aged 2 years) were used in the present study. All animals and experimental procedures were approved by the Northwest A&F University Institutional Animal Care and Use Committee. The sperm-rich fraction was collected weekly from each boar with gloved-hand technique and filtered using a double gauze.

### 3.3. Semen Processing

According to Okazaki et al. [30], the semen was directly diluted with Modena solution (*v* : *v* = 1 : 1) and incubated for 2 h at 15°C. The semen was divided into 6 parts, centrifuged for 10 min at 700 ×g to remove the Modena solution. The sperm pellets were resuspended with mNSF1 (2.0 × 10^9^ sperm/mL), added with different concentrations of resveratrol (0, 25, 50, 75, 100, or 125 *μ*M), and slowly cooled from 15 to 5°C over 1.5 h. Subsequently, the sperm suspension was diluted in the same volume of mNSF2 and packed into a 0.5 mL plastic straw. The straws were placed in liquid nitrogen vapor for 10 min and plunged into it for storage. The straws were stored in the liquid nitrogen at least one week. The straws were thawed in water at 60°C for 8 s, and the frozen-thawed sperm was quickly diluted with 4.5 mL of thawing solution.

### 3.4. Evaluation of Sperm Motility, Membrane Integrity, and Acrosome Integrity

Sperm motility was measured using a computer-assisted sperm motility analysis (CASA) system (Integrated Semen Analysis System; Hview, Fuzhou, China). Briefly, 5 *μ*L of semen was placed on an analyzer's Makler chamber and maintained at 37°C during the analysis. Three fields were selected for computer-assisted analysis [18].

Sperm membrane integrity and acrosome integrity were evaluated using a LIVE/DEAD Sperm Viability Kit (L7011; Thermo Fisher Scientific) and a fluorescein isothiocyanate-peanut agglutinin (FITC-PNA), respectively, according to Zhu et al. [8]. The stained sperm was monitored and photographed by an epifluorescence microscope (Nikon 80i; Tokyo, Japan) with a set of filters (200x).

### 3.5. Mitochondrial Activity

JC-1 Mitochondrial Membrane Potential Detection Kit (Beyotime Institute of Biotechnology, China) was used to analyze the changes in sperm mitochondrial activity (ΔΨm) [25, 31]. There are two types of JC-1 in stained mitochondrial plasma; one is a monomer that emits green fluorescence in a low ΔΨm, while the aggregates emit red fluorescence in a high ΔΨm. Briefly, sperm samples (2 × 10^6^/mL) were stained with 1x JC-1 at 37°C for 30 min. Fluorescence intensity of both mitochondrial JC-1 monomers (*λ*ex 514 nm, *λ*em 529 nm) and aggregates (*λ*ex 585 nm, *λ*em 590 nm) was detected using a monochromator microplate reader (Safire II, Tecan, Switzerland). The Δ*ψ*m of sperm in each treatment group was calculated as the fluorescence ratio of red (aggregates) to green (monomer). Analyses were performed in triplicate (*n* = 3).

### 3.6. Lipid Peroxidation

The probe BODIPY 581/591C_11_ (Molecular Probes) was used to measure the sperm lipid peroxidation according to our previous study [32]. The intact probe fluoresces red when it is intercalated into the membrane, and it shifts to green after oxidative radical attacked. The staining samples were analyzed using a fluorescence microreader with an emission filter set to 635 nm for red and 535 nm for green. All experiments were carried out in triplicate.

### 3.7. Detection of Sperm Oxidative DNA Damage

The generation of the oxidized base adduct, 8-hydroxyguanosine (8-OHdG), was detected as a biomarker for oxidative DNA damage. The detection of 8-OHdG in postthaw boar sperm was according to our previous study [32]. Sperm samples were washed twice and resuspended in 500 *μ*L PBS for flow cytometric analysis after 8-OHdG staining. BL2 (red fluorescence, long-pass dichroic filter 600 nm, bandpass filter 575 nm, detection width 560-590 nm) was used to detect the 8-OHdG level of postthaw sperm. A total of 20,000 sperm-specific events were evaluated. Data were processed by using the CellQuest program (BD Biosciences). The samples were also viewed and photographed using an epifluorescence microscope (80i; Nikon) with a set of filters (400x). Negative control with mouse IgG instead of the anti-8-OHdG antibody was included to ensure assay specificity.

### 3.8. Annexin V-FITC/PI Assay

Annexin V-FITC/PI apoptosis detection kit (Sigma-Aldrich, St. Louis, MO, USA) was used to assess sperm apoptosis according to the manufacturer's instruction with slight modifications. The postthaw sperm was centrifuged and washed thrice with PBS at 400 ×g for 5 min. The sperm was resuspended with 1x Annexin V-binding buffer at a concentration of 1 × 10^6^ sperm/mL. A total of 5 *μ*L Annexin V-FITC (AN) and 3 *μ*L PI were then added to each aliquot of a 100 *μ*L sample. The tubes were mixed gently and incubated at room temperature for 10 min in the dark. For the flow cytometric analysis, BL2 (red fluorescence, long-pass dichroic filter 600 nm, bandpass filter 575 nm, detection width 560-590 nm) and BL1 (green fluorescence, long-pass dichroic filter 550 nm, bandpass filter 525 nm, detection width 505-545 nm) were used to detect the stained sample. A total of 20,000 sperm-specific events were evaluated. Different labeling patterns of the stained sperm were also observed with a fluorescence microscope (80i; Nikon) at 400x magnification.

### 3.9. Detection of Sperm Intracellular ROS and GSH Level

Sperm ROS and GSH level were measured with Reactive Oxygen Species Assay Kit (Beyotime Institute of Biotechnology, China) and Cell Tracker Blue CMF_2_HC Molecular Probes (Invitrogen Inc., Carlsbad, CA, USA), respectively [31]. The sperm samples were incubated for 30 min in the Modena extender containing DCFH-DA (10 *μ*M) and Cell Tracker Blue (10 *μ*M), respectively. The samples were washed by centrifugation at 800 ×g for 5 min to remove the unbound probe and analyzed with a microplate reader (Synergy HT, BioTek, USA) at 485 nm excitation and 535 nm emission for ROS and 371 nm excitation and 464 nm emission for GSH. The stained samples were also viewed and photographed under a fluorescence microscope (80i; Nikon).

### 3.10. Measurement of GPx, SOD, and Catalase Activities

Activities of sperm GPx, SOD, and catalase were analyzed by the glutathione peroxidase assay kit, total superoxide dismutase assay kit, and catalase assay kit, respectively (Beyotime Institute of Biotechnology, China), according to Zhu et al. [32, 33]. The sperm pellets were rinsed three times with PBS and resuspended, then lysed ultrasonically (20 kHz, 750 W, operating at 40%, on 3 s, off 5 s, 5 cycles) on ice and centrifuged at 12,000 ×g for 10 min at 4°C. The supernatants were used to analyze the GPx, SOD, and catalase activities according to the manufacturer's instruction.

### 3.11. Immunofluorescence

Postthaw sperm was fixed with 4% paraformaldehyde for 10 min at room temperature after washing in PBS. The sperm sample was spread onto the poly-L-lysine slides and air dried at room temperature. The samples were permeabilized with 0.5% Triton X-100 in PBS for 10 min. Nonspecific binding was blocked with PBS supplementation of 10% BSA for 30 min at room temperature. Samples were then incubated overnight at 4°C with anti-AMPK (1 : 100, CST). On the next day, the sperm were washed three times in PBS and incubated with the goat anti-rabbit (1 : 100, Santa Cruz Biotechnology) antibody for immunofluorescence labeling. Sperm was washed and counterstained with DAPI (CWBIO); fluorescent images were captured with fluorescence microscopy (80i, Nikon).

### 3.12. Western Blotting

Sperm total protein was extracted according to our previous study [25]; 25 *μ*g of the extracted protein was added to each lane of a 12.5% polyacrylamide gradient gel, then transferred to the PVDF membrane. After being blocked with 5% BSA, the membrane was incubated with anti-*α*-tubulin (Santa Cruz, 1 : 1000), anti-AMPK (CST, 1 : 1000), anti-p-AMPK (CST, 1 : 1000), anti-p53 (CST, 1 : 1000), anti-cleaved caspase-3 (CST, 1 : 1000), anti-cleaved caspase-9 (CST, 1 : 1000), and anti-Parp-1 (CST, 1 : 1000) at 4°C overnight and incubated with the secondary antibody (CWBio, 1 : 2000) at room temperature for 1 h. Enhanced chemiluminescence (ECL) detection was performed by using the ECL™ Prime Western Blotting Detection Reagents (RPN2235, GE Bioscience) according to the manufacturer's specifications and appropriate exposure of blots to Fuji X-ray film (ChampChemi Top 610, China).

### 3.13. Statistical Analysis

All data were analyzed by one-way ANOVA, and Tukey's multiple comparison test was performed using SPSS version 19.0 for Windows (SPSS Inc., Chicago, IL). All values are presented as mean ± standard error of the mean (SEM). Differences with values of *p* < 0.05 considered to be statistically significant.

## 4. Result

### 4.1. Resveratrol Improved Sperm Motility Patterns, Membrane Integrity, and Acrosome Integrity during Cooling, Freezing, and Thawing Processes

**Figure 1:**
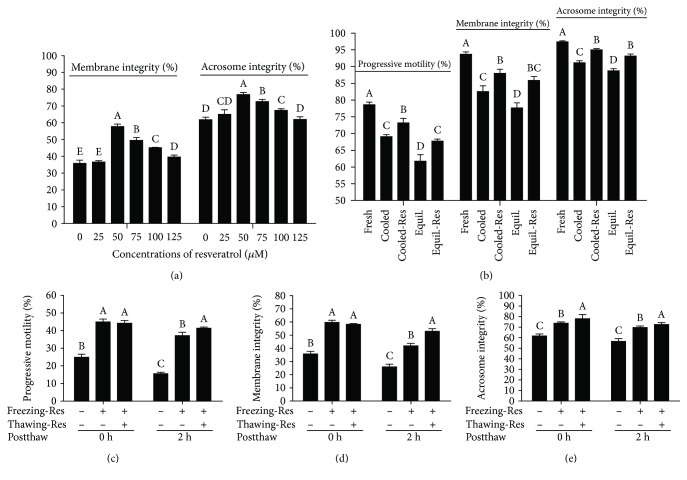
Effects of resveratrol on sperm progressive motility, membrane integrity, and acrosome integrity during cooling, freezing, and thawing incubation processes. Cooled: fresh sperm cooled from room temperature to 5°C. Cooled-Res: fresh sperm cooled from room temperature to 5°C with 50 *μ*M resveratrol. Equil.: cooled sperm equilibrated for 30 min at 5°C. Equil.-Res: cooled sperm equilibrated for 30 min at 5°C with 50 *μ*M resveratrol. Freezing-Res: freezing extender added with (+) or without (-) 50 *μ*M resveratrol. Thawing-Res: thawing solution supplemented with (+) or without (-) 50 *μ*M resveratrol. Data are the mean ± SEM (*n* = 5 independent replicates). Columns with different uppercase letters differ significantly (*p* < 0.05).

Compared to the control, the values of postthaw sperm total motility (TM), progressive motility (PM), straight-line velocity (VSL), linearity (LIN), curvilinear velocity (VCL), and average path velocity (VAP) were significantly improved by the addition of resveratrol (from 50 to 125 *μ*M). Meanwhile, the beat-cross frequency (BCF) was unchanged in all treatments (Table 1). Moreover, the membrane integrity and acrosome integrity of postthaw sperm were also significantly increased by resveratrol treatment with all doses from 50 to 125 *μ*M (Figure 1(a)). Interestingly, the 50 *μ*M dose of resveratrol showed the highest value in those parameters (Table 1 and Figure 1(a)).

To elucidate whether the resveratrol could improve the sperm quality at each step of cryopreservation and postthaw incubation, the boar sperm was exposed with 50 *μ*M resveratrol during the cryopreservation processes and postthaw incubation. It was observed that the progressive motility, membrane integrity, and acrosome integrity were significantly increased in resveratrol-treated sperm during cooling from room temperature to 5°C and equilibration at 5°C for 30 min (Figure 1(b)) and also during the 2 h of postthaw incubation (Figures 1(c)–1(e)).

### 4.2. Resveratrol Reduced the Lipid Peroxidation and Oxidative DNA Damage and Increased the Mitochondrial Activity of Postthaw Sperm

**Figure 2:**
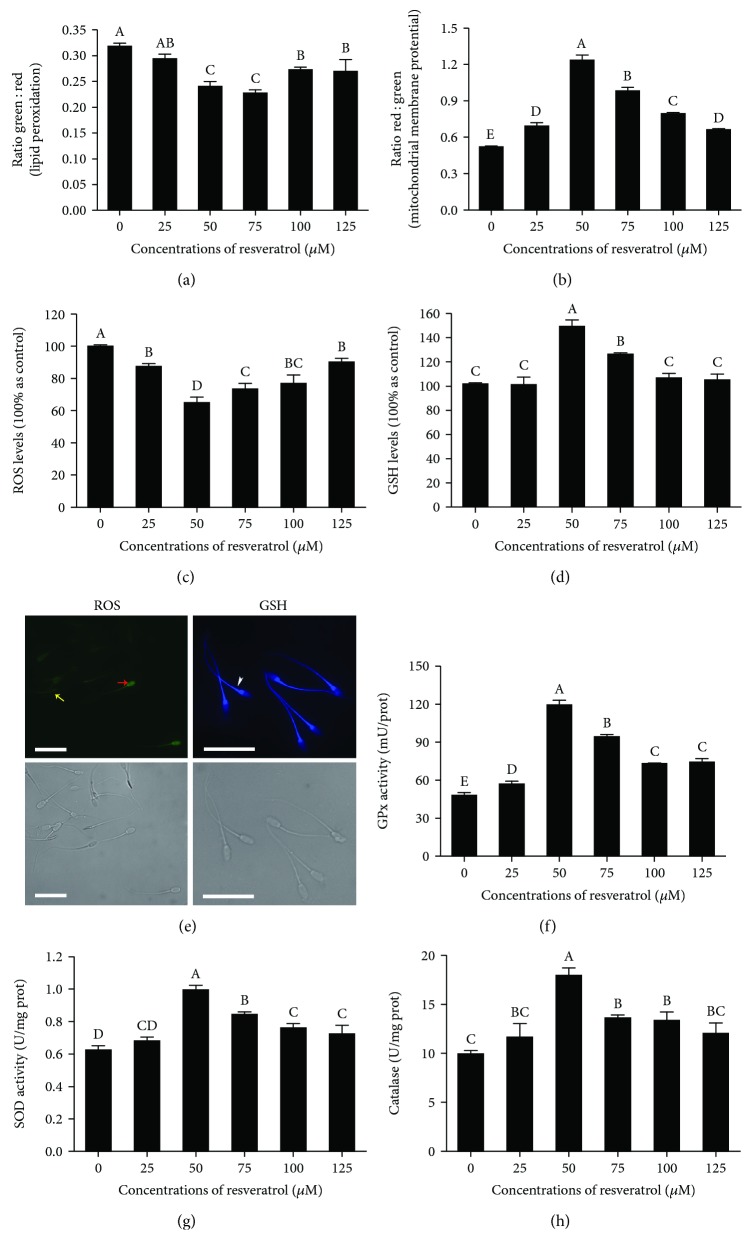
Effects of different concentrations of resveratrol on postthaw sperm lipid peroxidation (a), mitochondrial membrane potential (b), ROS level (c), GSH level (d), GPx activity (f), SOD activity (g), and catalase activity (h). Photomicrographs of the postthaw sperm stained with ROS and GSH probes, respectively (e): the red arrow indicates sperm with a high level of intracellular ROS (high green fluorescence level), the yellow arrow indicates sperm with a low intracellular ROS level (low green fluorescence level), and white arrow indicates the distribution of GSH in sperm. Bars = 30 *μ*m.

**Figure 3:**
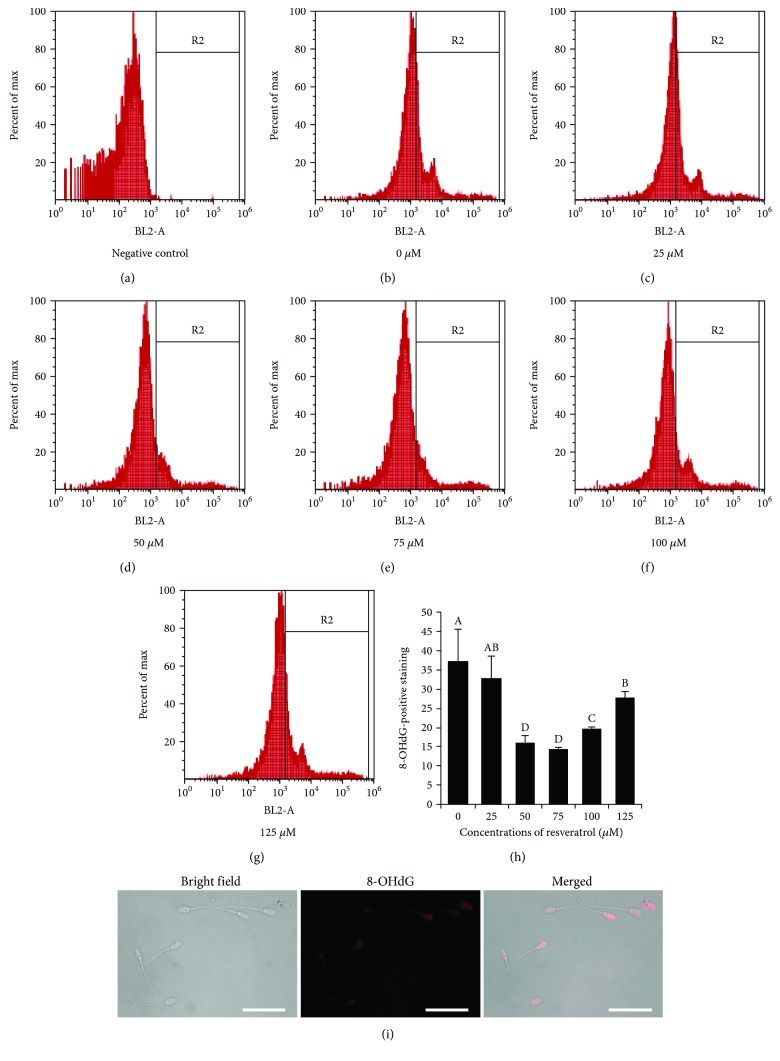
Effects of different concentrations of resveratrol on sperm oxidative DNA damage (b–h). Negative control (a). Photomicrographs of the postthaw sperm stained with 8-OHdG (i). Columns with different uppercase letters differ significantly (*p* < 0.05). Bars = 30 *μ*m.

The lipid peroxidation of postthaw sperm was significantly decreased in the treatments with 50, 75, 100, and 125 *μ*M resveratrol; meanwhile, the 50 and 75 *μ*M resveratrol treatments showed the lowest lipid peroxidation (Figure 2(a)). Interestingly, the mitochondrial activity was significantly increased by adding resveratrol, especially 50 *μ*M to the freezing medium (Figure 2(b)). Moreover, the analysis of the oxidative DNA damage in postthaw sperm using 8-hydroxydeoxyguanosine (8-OHdG) staining revealed that the level of 8-OHdG in postthaw sperm was significantly decreased with the addition of 50, 75, 100, and 125 *μ*M resveratrol (Figures 3(a)–3(h)). In addition, the 8-OHdG was also detected in the head and midpiece of sperm, where the sperm nuclear DNA and mitochondrial DNA are located, respectively (Figure 3(i)).

### 4.3. Resveratrol Decreased the ROS Level, Increased the Activities of GPx, SOD, and CAT, and Maintained GSH Content of Postthaw Sperm

The ROS level of postthaw sperm was significantly decreased by the addition of resveratrol to the freezing medium, where the 50 *μ*M resveratrol treatment showed the lowest ROS level (Figure 2(c)). The sperm with a high green fluorescence level indicated that the sperm was in a high level of intracellular ROS (Figure 2(e), red arrow), whereas the low green fluorescence indicated sperm with low intracellular ROS (Figure 2(e), yellow arrow). Moreover, the addition of 50 and 75 *μ*M resveratrol significantly increased the GSH level, compared to the control (Figure 2(d)). It was observed that the GSH was distributed in the sperm head, midpiece, and tail, and higher blue fluorescence in the tail, midpiece, and postacrosomal sheath of the sperm head indicated that the GSH level was higher in those sections (Figure 2(e), white arrow).

The GPx, SOD, and catalase enzymes are components of the cellular antioxidative defense system. It was observed that the addition of resveratrol to the freezing medium increased the activities of GPx, SOD, and catalase in postthaw sperm (Figures 2(f)–2(h)). Additionally, the postthaw sperm treated with 50 *μ*M resveratrol presented the highest value of those enzyme activities among all the treatments (Figures 2(f)–2(h)).

### 4.4. Resveratrol Promotes the AMPK Phosphorylation against the ROS Damage for Improving Sperm Quality

**Figure 4:**
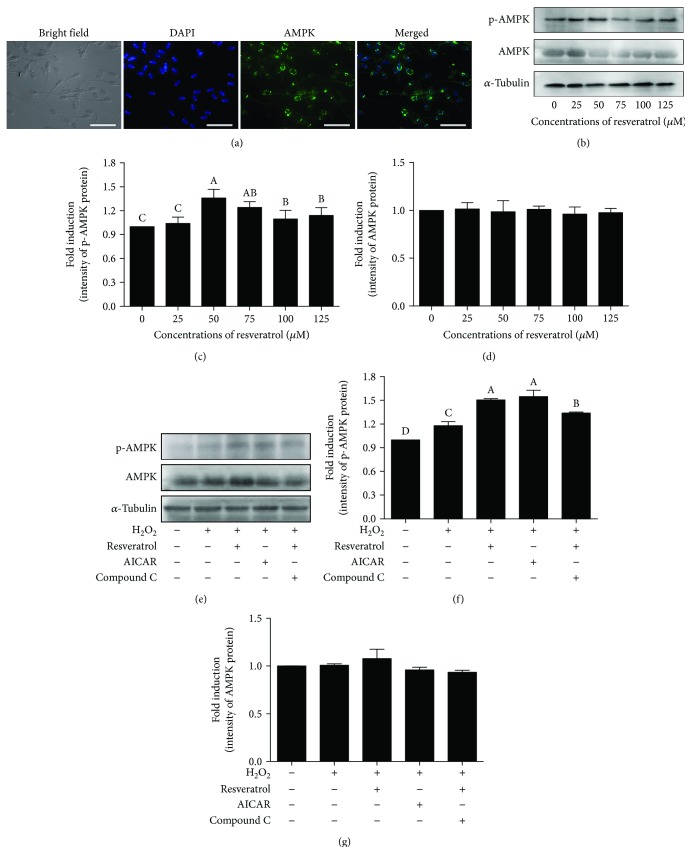
Location of AMPK in postthaw boar sperm was analyzed by immunofluorescence (a). Effects of different concentrations of resveratrol on postthaw boar sperm AMPK phosphorylation (b–d). (b) Western blotting image is showing the expression of the p-AMPK, AMPK, and *α*-tubulin of postthaw boar sperm. (c, d) Quantitative expression of the p-AMPK and AMPK over *α*-tubulin generated from western blotting (b). (e) Western blotting image is showing the expression of the p-AMPK, AMPK, and *α*-tubulin of sperm in the H_2_O_2_-induced oxidative stress model. (f, g) Quantitative expression of the p-AMPK and AMPK over *α*-tubulin generated from western blotting (e). Data are the mean ± SEM (*n* = 3 independent replicates). Columns with different uppercase letters differ significantly (*p* < 0.05).

To investigate the mechanism of resveratrol on how to improve sperm quality, the AMPK phosphorylation of postthaw sperm was detected. The AMPK protein was localized in the acrosome and the midpiece of the flagellum in postthaw boar sperm (Figure 4(a)). The addition of 50, 75, 100, and 125 *μ*M resveratrol significantly increased the Thr^172^-AMPK phosphorylation of postthaw sperm; among all doses, the 50 *μ*M treatments showed the highest value of Thr^172^-AMPK (Figures 4(b)–4(d)). However, there is no significant change in the total AMPK level among all the treatments (Figures 4(b)–4(d)). To investigate whether the resveratrol protects boar sperm via activating AMPK against ROS stress *in vitro*, we used H_2_O_2_ to induce the ROS damages, as well as the AMPK activator (5-aminoimidazole-4-carboxamide-1-beta-4-ribofuranoside (AICAR)), and inhibitor (Compound C) was used to regulate AMPK. As showed in Figures 4(e)–4(g), the H_2_O_2_ treatment promoted the sperm AMPK phosphorylation compared to the control. It was also observed that the addition of either resveratrol or AICAR to the H_2_O_2_ treatment significantly increased the AMPK phosphorylation; however, the addition of Compound C counteracted the effect of resveratrol (Figures 4(e) and 4(f)). The total level of AMPK protein was observed to remain unchanged in all treatments (Figure 4(g)).

**Figure 5:**
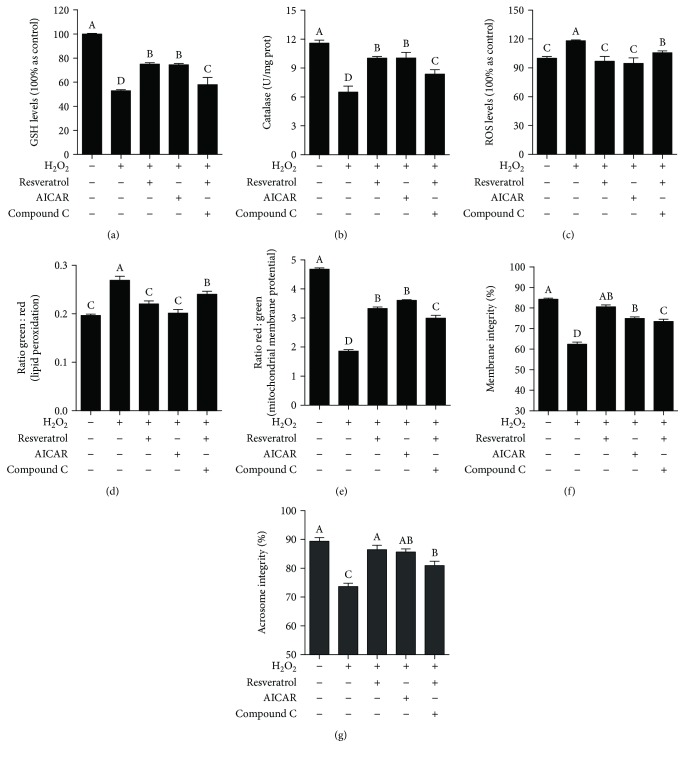
Effects of resveratrol, AMPK activator (AICAR), and inhibitor (Compound C) on the sperm GSH level (a), catalase activity (b), ROS level (c), lipid peroxidation (d), mitochondrial membrane potential (e), membrane integrity (f), and acrosome integrity (g) in the H_2_O_2_-induced oxidative stress model *in vitro*. Data are the mean ± SEM (*n* = 3 independent replicates). Columns with different uppercase letters differ significantly (*p* < 0.05).

Moreover, the GSH content and catalase activity of sperm were observed to be significantly decreased when incubated with H_2_O_2_ compared to control, which were significantly improved by treatment with either resveratrol or AICAR (Figures 5(a) and 5(b)). However, the positive effects of resveratrol on the sperm antioxidative defense system were counteracted with the addition of Compound C (Figures 5(a) and 5(b)). The sperm ROS level and lipid peroxidation were also reduced in either resveratrol or AICAR treatment (Figures 5(c) and 5(d)). Furthermore, sperm parameters (such as sperm motility patterns, mitochondrial activity, membrane integrity, and acrosome integrity) were also improved by the treatment with resveratrol or AICAR compared to the H_2_O_2_ treatment (Table 2 and Figures 5(e)–5(g)).

### 4.5. Resveratrol Decreased the Postthaw Boar Sperm Apoptosis

**Figure 6:**
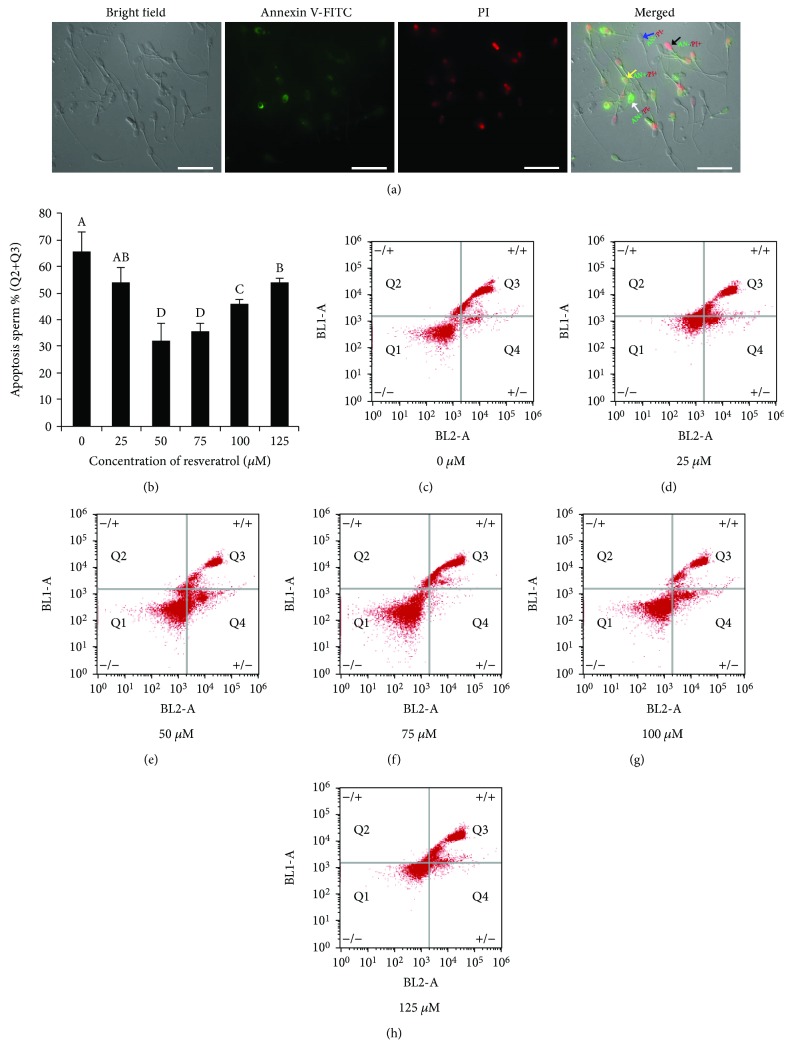
Photomicrographs of the postthaw sperm stained with the Annexin V-FITC/PI assay kit: live sperm (AN-/PI-; blue arrow), early apoptotic sperm (AN+/PI-; white arrow), late apoptotic sperm (AN+/PI+; yellow arrow), and nonviable necrotic sperm (AN-/PI+; black arrow) (a). Effects of different concentrations of resveratrol on postthaw sperm apoptosis (b–h). Data are the mean ± SEM (*n* = 3 independent replicates). Columns with different uppercase letters differ significantly (*p* < 0.05).

**Figure 7:**
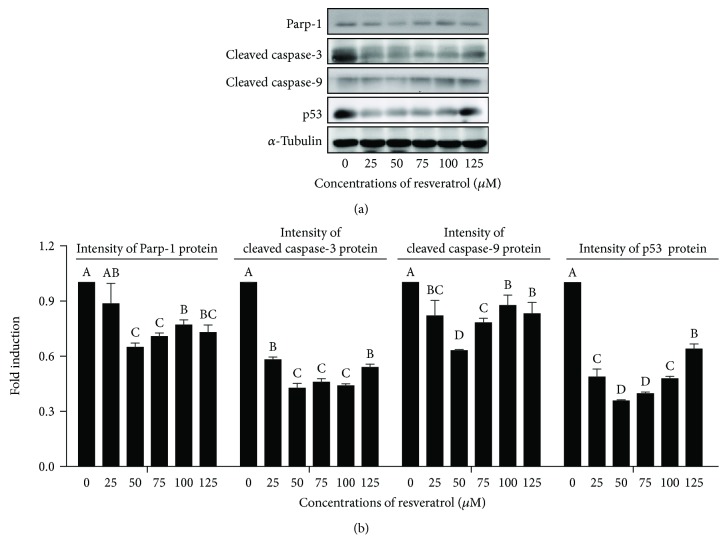
Western blotting image is showing the expression of apoptosis proteins in postthaw boar sperm (a). (b) Quantitative expression of the Parp-1, cleaved caspase-3, cleaved caspase-9, and p53 over *α*-tubulin generated from western blotting. Data are the mean ± SEM (*n* = 3 independent replicates). Columns with different uppercase letters differ significantly (*p* < 0.05).

As shown in Figure 6(a), the postthaw sperm stained with the Annexin V-FITC/PI assay kit were observed with following four subpopulations: live sperm (AN-/PI-; blue arrow), early apoptotic sperm (AN+/PI-; white arrow), late apoptotic sperm (AN+/PI+; yellow arrow), and nonviable necrotic sperm (AN-/PI+; black arrow). It was observed that the addition of resveratrol significantly decreased the apoptosis of postthaw sperm (Q2+Q3; Figures 6(b)–6(h)), and the 50 *μ*M resveratrol treatment showed the lowest percentage of postthaw sperm with apoptosis (Figures 6(b)–6(h)). Moreover, in western blotting, it was observed that the expression of apoptotic factor proteins (Parp-1, cleaved caspase-3, cleaved caspase-9, and p53) was significantly decreased by resveratrol treatments at different concentrations (25 to 125 *μ*M). Interestingly, the 50 *μ*M showed the lowest expression of the aforementioned apoptotic factor proteins (Figures 7(a) and 7(b)).

## 5. Discussion

Cryopreservation of sperm is an efficient procedure for the management and preservation of male fertility in human and domestic animals [34, 35]. Sperm cryopreservation is routinely used in the cases of azoospermia patients or the patients susceptible to infertility due to therapeutic treatments for malignant diseases, who want to have babies using their sperm at a later time [34]. In domestic animals, sperm cryopreservation is also an extensively practiced technique to accelerate the rate of genetic improvement [35]. However, during the cryopreservation, sperm mitochondrial dysfunction occurs due to suffering from sudden temperature changes, ice formation, and osmotic stress [36]. It is well known that most of the ROS are generated as a by-product in the cellular mitochondrial oxidative phosphorylation of the energy pathway [19]. Moreover, the sperm ROS level was observed to be significantly increased during the cryopreservation process in our previous study [8]. The excess ROS reduced the fertilization by decreasing sperm quality [37]. In this study, the sperm quality was reduced with the increase of the ROS level during cooling, freezing, and thawing processes. The supplementation of resveratrol, a mitochondria-targeted antioxidant, significantly improved sperm progressive motility, membrane integrity, acrosome integrity, mitochondrial activity, and AMPK phosphorylation as well as the sperm antioxidative defense system and reduced the ROS level, lipid peroxidation, and sperm apoptosis, suggesting that the resveratrol is beneficial for improving the quality of postthaw boar sperm by activating the AMPK activity to reduce sperm apoptosis.

Cellular ROS homeostasis is controlled by the ROS generation and elimination in cells [38]. The mitochondria are the main ROS-generated site in sperm [39]. The emission of electrons from the mitochondrial electron transfer chain forms the ROS, which was reported to be increased with the mitochondrial dysfunction during cryopreservation [40, 41]. In addition, the cellular antioxidative systems (scavenging enzymes) are located in the cytoplasm and the sperm contain very few cytoplasms [42], indicating that sperm is susceptible to ROS damage. Cryopreservation also has been shown to diminish the sperm antioxidant enzyme activity [43, 44], suggesting that the ability of sperm to scavenge ROS is reduced during the cryopreservation process. The peroxidative damage induced by increased concentration of ROS is associated with the damage to the membrane integrity, acrosome integrity, and DNA stability as well as mitochondrial function [45], which lead to induced sperm apoptosis and ultimately reduced fertility [46]. Therefore, reducing the ROS generation, as well as enhancing the ROS scavenging ability, is essential to minimize the damage of oxidative stress in sperm.

AMPK is a key kinase involved in regulating the cellular redox state by switching the metabolic pathway under the stressful conditions [47]. In somatic cells, AMPK could promote glucose uptake and glycolysis, facilitating antioxidant production [47]. Kukidome et al. reported that the activation of AMPK by AICAR reduced the hyperglycemia-induced mitochondrial ROS generation in human umbilical vein endothelial cells [48]. AMPK activation by metformin also suppressed ROS production in mouse Schwann cells [49]. Moreover, Kim et al. showed that AMPK activation could inhibit palmitate-induced apoptosis through suppression of ROS production in bovine aortic endothelial cells [50]. *In vivo* AMPK activation also decreased the ROS level in rat diabetic fibrosis, kidney tissues, and type 2 diabetes patients [51, 52]. Additionally, AMPK activation has been reported to increase the expression of antioxidant enzymes in monocytes-macrophages [53] and restore GSH depletion [52]. Furthermore, in chicken and rabbit sperm cryopreservation, AMPK activation by either AICAR or metformin significantly increased the SOD, GPx, and catalase activities of postthaw sperm, whereas the Compound C decreased those enzyme activities and increased the level of lipid peroxidation and ROS with negative effects on the sperm quality [31, 54]. Therefore, if the AMPK is activated during the cryopreservation, the quality of postthaw sperm will be improved through the AMPK regulating ROS generation and sperm antioxidative defense.

Notably, it has been reported that the AMPK was activated by the addition of resveratrol in liver steatosis of either different hepatic cell models *in vitro* or animal models (mice and rats) *in vivo* [55]. Moreover, the activation of AMPK by resveratrol in somatic cells was revealed through a variety of mechanisms in previous studies [56, 57]. In the present study, it was observed that the AMPK phosphorylation of postthaw boar sperm was significantly increased by the addition of 50 *μ*M resveratrol to the freezing extender. The antioxidative defense (GSH level and activities of GPx, SOD, and catalase) in postthaw sperm was also increased with the treatment with 50 *μ*M resveratrol, while the ROS level was decreased. Interestingly, in the H_2_O_2_-induced sperm ROS damage model, the positive effects of resveratrol were partly counteracted in the presence of the AMPK inhibitor (Compound C). These observations suggested that the addition of resveratrol could decrease ROS production and enhance the antioxidative defense of postthaw boar sperm by promoting AMPK phosphorylation.

Resveratrol, a mitochondria-targeted antioxidant, has been not only used as a therapy to human aging disease *in vivo* but also used to improve male fertility *in vitro* and *in vivo* [58]. Bucak et al. reported that the addition of 1 mM resveratrol to the freezing medium significantly improved postthaw bull sperm motility and mitochondrial activity [26]. Moreover, the resveratrol also decreased the lipid peroxidation as well as DNA damage of postthaw sperm in bull [26] and human [27]. In the present study, it was also observed that the addition of 50 *μ*M resveratrol improved postthaw boar sperm motility, membrane integrity, acrosome integrity, and DNA stability as well as mitochondrial activity. These observations coincided with the changes in the ROS level and antioxidative defense after treatment with resveratrol. The high concentration of ROS and fall of antioxidant enzymes led to cell apoptosis [59]. The addition of resveratrol significantly reduced sperm apoptosis in this study, suggesting that the resveratrol might decrease the postthaw sperm apoptosis via scavenging the excess ROS and enhancing the sperm antioxidative defense system. Therefore, the addition of resveratrol is beneficial for improving sperm quality during cryopreservation.

In conclusion, the ROS is induced in sperm during cryopreservation. The addition of resveratrol activates AMPK phosphorylation, which reduces the ROS production and enhances the sperm antioxidative defense system (such as the GSH level and activities of GPx, SOD, and catalase). Consequently, the ROS level was decreased, and thereby, the sperm motility, membrane integrity, and acrosome integrity as well as mitochondrial activity were increased while reducing the sperm apoptosis and DNA damage. This study using the boar sperm cryopreservation model helps us to understand whether and how the resveratrol improves sperm quality, which will contribute new insights to the human and animal reproductive field.

## Figures and Tables

**Table 1:** Effects of different concentrations of resveratrol on postthaw sperm motility parameters.

Sperm parameters	Resveratrol (*μ*M)					
	0	25	50	75	100	125
TM (%)	39.7 ± 3.5^c^	38.6 ± 3.5^c^	64.3 ± 2.2^a^	60.7 ± 3.4^a^	49.3 ± 3.3^b^	44.7 ± 3.7^bc^
PM (%)	24.7 ± 1.5^c^	24.2 ± 2.5^c^	45.5 ± 3.1^a^	42.6 ± 3.0^a^	33.5 ± 2.0^b^	27.5 ± 6.8^bc^
LIN (%)	23.4 ± 2^d^	26.1 ± 1.6^c^	33.5 ± 1.3^a^	30.2 ± 1.0^b^	30.3 ± 1.4^b^	26.9 ± 0.9^c^
VSL (*μ*m/s)	20.9 ± 2.7^a^	28.5 ± 4.1^bc^	44.2 ± 1.9^a^	41.4 ± 2.9^a^	36.5 ± 2.1^b^	33.0 ± 3.3^b^
VCL (*μ*m/s)	90.1 ± 5.4^c^	103.7 ± 9.5^bc^	133.1 ± 6.0^a^	134.8 ± 5.1^a^	124.5 ± 6.3^b^	124.9 ± 7.0^b^
VAP (*μ*m/s)	36.9 ± 4.4^d^	48.1 ± 5.4^c^	69.4 ± 3.0^a^	67.3 ± 3.6^a^	60.0 ± 3.1^b^	59.9 ± 5.9^b^
BCF (Hz)	26.7 ± 0.9^a^	28.3 ± 1.8^a^	27.3 ± 0.5^a^	27.9 ± 0.2^a^	29.9 ± 2.9^a^	28.4 ± 1.5^a^

Values are expressed as mean ± SEM. Different letters within column indicate significant difference (*p* < 0.05). TM: total motility; PM: progressive motility; VCL: curvilinear velocity; VSL: straight-line velocity; VAP: average path velocity; BCF: beat-cross frequency; LIN: linearity (VSL/VCL).

**Table 2:** Effects of resveratrol, AMPK activator (AICAR), and inhibitor (Compound C) on sperm motility parameters in the H_2_O_2_-induced oxidative stress model *in vitro*.

Sperm parameters	FS	H_2_O_2_	H_2_O_2_+R	H_2_O_2_+AICAR	H_2_O_2_+R+CC
TM (%)	80.9 ± 6.5^a^	58.5 ± 9.6^c^	64.3 ± 2.2^b^	68.4 ± 8.8^b^	65.5 ± 9.0^b^
PM (%)	62.8 ± 5.2^a^	38.9 ± 4.1^d^	52.7 ± 2.0^b^	53.3 ± 2.6^b^	46.5 ± 5.9^c^
LIN (%)	46.8 ± 2.5^a^	37.7 ± 3.5^c^	43.4 ± 1.4^a^	43.6 ± 1.1^a^	44.9 ± 3.4^a^
VSL (*μ*m/s)	80.3 ± 4.4^a^	56.4 ± 8.8^d^	73.2 ± 4.3^b^	72.8 ± 6.1^b^	64.0.5 ± 6.4^c^
VCL (*μ*m/s)	171.9 ± 3.4^a^	136.3 ± 14.2^b^	169.3 ± 11.4^a^	165.5 ± 11.0^a^	167.9 ± 8.4^a^
VAP (*μ*m/s)	101.9 ± 1.9^a^	82.0 ± 19.7^c^	95.5 ± 5.6^ab^	94.1 ± 5.3^ab^	91.6 ± 4.2^b^
BCF (Hz)	30.9 ± 1.0^a^	30.9 ± 3.0^a^	28.0 ± 2.7^a^	30.9 ± 0.9^a^	29.1 ± 2.0^a^

Values are expressed as mean ± SEM. Different letters within the column indicate significant difference (*p* < 0.05). TM: total motility; PM: progressive motility; VCL: curvilinear velocity; VSL: straight-line velocity; VAP: average path velocity; BCF: beat-cross frequency; LIN: linearity (VSL/VCL); FS: fresh sperm; R: resveratrol; CC: Compound C.

## Data Availability

All data used to support the findings of this study are included in the article.

## Abbreviations

AICAR: 5-Aminoimidazole-4-carboxamide-1-beta-4-ribofuranoside
AMPK: 5′AMP-activated protein kinase
ATP: Adenosine triphosphate
ROS: Reactive oxygen species
BCF: Beat-cross frequency
CASA: Computer-assisted sperm motility analysis
FITC-PNA: Fluorescein isothiocyanate-peanut agglutinin
GPx: Glutathione peroxidase
GSH: Glutathione
LIN: Linearity
NSF: Freezing extender
OXPHOS: Mitochondrial oxidative phosphorylation
PM: Progressive motility
SEM: Standard error of the mean
SOD: Superoxide dismutase
TM: Total motility
VAP: Average path velocity
VSL: Straight-line velocity
VCL: Curvilinear velocity
ΔΨm: Mitochondrial activity
8-OHdG: 8-Hydroxyguanosine.

## Supplementary Materials

Supplementary Materials Supplementary Figure 1 Group (1): Modena extender containing 200 *μ*M H_2_O_2_. Group (2): Modena extender containing 200 *μ*M H_2_O_2_ and 50 *μ*M resveratrol. Group (3): Modena extender containing 200 *μ*M H_2_O_2_ and 2 mM AICAR. Group (4): Modena extender containing 200 *μ*M H_2_O_2_, 50 *μ*M resveratrol, and 30 *μ*M Compound C. Group (5): Modena extender without resveratrol, AICAR, Compound C, or H_2_O_2_.
