# Supplementary material for: Resveratrol Improves Boar Sperm Quality via 5′AMP-Activated Protein Kinase Activation during Cryopreservation
Source: Oxid Med Cell Longev. 2019 Sep 4;2019:5921503. doi: 10.1155/2019/5921503 (PMC6746164; doi:10.1155/2019/5921503)
Supplement: Supplementary Materials — Supplementary Figure 1 Group (1): Modena extender containing 200 μM H2O2. Group (2): Modena extender containing 200 μM H2O2 and 50 μM resveratrol. Group (3): Modena extender containing 200 μM H2O2 and 2 mM AICAR. Group (4): Modena extender containing 200 μM H2O2, 50 μM resveratrol, and 30 μM Compound C. Group (5): Modena extender without resveratrol, AICAR, Compound C, or H2O2. [file 5921503.f1.pdf]

Supplementary Figure

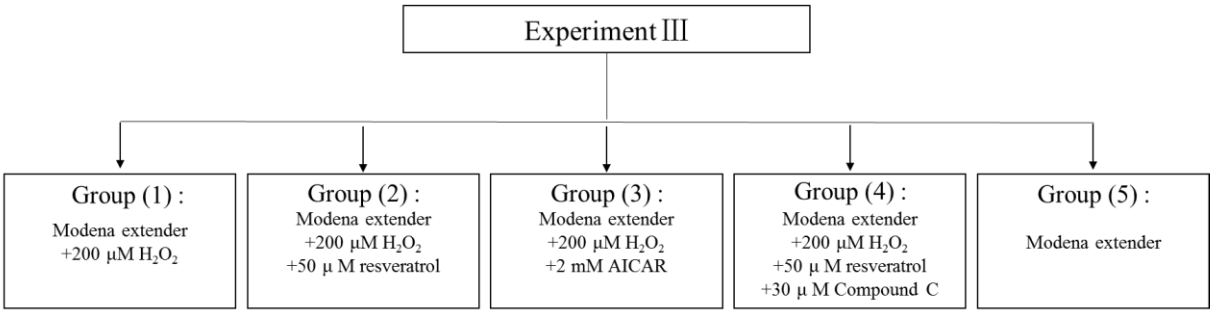

The scheme showed the five treatment groups in Experiment III

Supplementary Figure 1

Supplementary Figure 1: Group (1): Modena extender containing 200 μM H_2O_2; Group (2): Modena extender containing 200 μM H_2O_2 and 50 μM resveratrol; Group (3): Modena extender containing 200 μM H_2O_2 and 2 mM AICAR; Group (4): Modena extender containing 200 μM H_2O_2, 50 μM resveratrol and 30 μM Compound C; and Group (5): Modena extender without resveratrol, AICAR, Compound C or H_2O_2.
